# Depth of Invasion as a Predictor of Cervical Nodal Metastasis of Oral Tongue Squamous Cell Carcinoma: Findings From a Tertiary Care Center in Pakistan

**DOI:** 10.7759/cureus.18976

**Published:** 2021-10-22

**Authors:** Adnan Y Muhammad, Rahim Dhanani, Summaiya Salman, Zeeshan Shaikh, Shayan K Ghaloo, Mubasher Ikram

**Affiliations:** 1 ENT and Head & Neck Surgery, Hayatabad Medical Complex, Peshawar, PAK; 2 Otolaryngology - Head and Neck Surgery, Dr. Ziauddin University & Hospital, Karachi, PAK; 3 ENT, Sohail University, Karachi, PAK; 4 Surgery, Aga Khan University & Hospital, Karachi, PAK

**Keywords:** cervical nodes, squamous cell carcinoma, oral tongue, occult neck metastasis, depth of invasion

## Abstract

Background and objective

Cervical lymph node metastasis has a significant impact on the survival of patients with oral cavity tumors. The rate of occult neck node metastasis is reported to range from 20 to 40%. The depth of invasion (DOI) has been incorporated in the eighth edition of the American Joint Commission on Cancer (AJCC) staging manual and is an important predictor of cervical lymph node metastasis. In this study, we aimed to identify the occult neck node metastasis rate in early oral tongue squamous cell carcinoma (OTSCC) and correlate it with the DOI.

Methods

A retrospective review of all patients presenting to our facility with early-stage OTSCC was performed. Patients with tumor size of ≤4 cm and who underwent elective neck dissection at the time of surgery were included. The study outcomes were the rates of occult neck metastases in T1 and T2 OTSCC and their correlation with the DOI.

Results

There were 80 patients in total. Occult neck node metastases were seen in 29 (36.25%) patients. Patients with a DOI >5 mm were 1.41 times more likely to have occult neck node metastasis than those with a DOI ≤5 mm.

Conclusion

Occult neck node metastasis is significantly associated with the DOI. The risk of neck metastasis is higher in patients with a DOI >5 mm.

## Introduction

Oral tongue squamous cell carcinoma (OTSCC) is the most common cancer of the oral cavity, accounting for up to 25-40% of its malignancies [1,2]. Its incidence has been rising, especially in young adults, which has led to significant morbidity and mortality [3,4]. The risk factors include chewable tobacco, alcohol consumption, and the human papillomavirus (HPV) [5].

One of the most important factors affecting survival in oral cavity tumors is neck nodal metastatic disease. The risk of occult neck node metastasis in early-stage squamous cell carcinoma is reported to be up to 40%. Elective neck dissection (END) becomes mandatory when the risk of cervical metastasis is more than 15% [6]. In patients in whom only primary resection is done, with the watchful observation of the neck during follow-up, the relapsed cervical disease might present a challenge due to the high rates of extracapsular spread. However, some surgeons consider performing neck dissection for early-stage tumors as overtreatment [6].

The eighth edition of the staging manual of The American Joint Commission on Cancer (AJCC) includes the depth of invasion (DOI) in the T classification [7]. The DOI has been an important factor in reshaping the staging system, with the tumor stage upgraded based on DOI cutoff values of 5 and 10 mm [7]. Our study aimed to identify the rate of occult neck node metastasis in early OTSCC and correlate it with the DOI.

## Materials and methods

After receiving an exemption from the Institutional Review Board (IRB) (3408-Sur-ERC-14), a retrospective review was conducted at the Department of Otolaryngology-Head and Neck Surgery at the Aga Khan University Hospital in Karachi, Pakistan. The medical records of all patients presenting to the facility from April 2000 to March 2010 with a diagnosis of OTSCC were reviewed.

Patients with a tumor of ≤4 cm who underwent END at the time of primary surgery were included in the study. Patients who underwent surgery outside our center, those with T3 or T4 tumors, those with recurrent disease, those receiving neoadjuvant therapy, those with cervical or distant metastases, and patients with missing data were excluded. Cervical lymph node metastasis was assessed by clinical examination and radiological investigation (CT or MRI scan).

As recommended in the eighth edition of the AJCC cancer staging manual [7], the DOI was measured by a plumb line drawn from the basement membrane of the nearest normal mucosa to the deepest point of invasion. All specimens were reported by a head and neck pathologist, who was blinded to the patients’ clinical data and outcomes. Based on previously published data, patients were divided into two groups according to the DOI: ≤5 mm and >5 mm [6,8,9].

We recorded age, gender, tumor size, DOI, and nodal metastasis. The study outcomes were the rates of occult neck metastasis in T1 and T2 OTSCC and their correlation with the DOI. The data was recorded and analyzed with SPSS Statistics version 25.0 (IBM, Armonk, NY). The Chi-square test was applied to examine the correlation of occult nodal metastasis with the DOI, and relative risk was calculated.

## Results

A total of 312 patients who were treated at our center with OTSCC over 10 years were identified; 80 patients who fulfilled the inclusion criteria were included in the study. The mean age of the patients was 49.7 ±14.1 years (range: 20-90 years). There were 49 (61.25%) male patients and 31 (38.75%) female patients. Tobacco chewing and smoking were the most common risk factors identified in our patients. The patient characteristics are shown in Table 1.

**Table 1:** Patient and tumor characteristics SD: standard deviation; DOI: depth of invasion; T: tumor; N: nodal

Characteristics		
	Mean ±SD, range	
Age, years	49.7 ±14.1, 20–90	
DOI, mm	5.1 ±2.2, 1–9	
	Frequency (n)	Percentage (%)
Gender		
Male	49	61.25%
Female	31	38.75%
Risk factors		
Tobacco chewing	20	25%
Smoking	12	15%
Smoking + tobacco chewing	9	11.25%
Alcohol consumption	2	2.5%
Tumor grade		
Well-differentiated	29	36.25%
Moderately differentiated	47	58.75%
Poorly differentiated	4	5%
T stage		
T1	34	42.5%
T2	46	57.5%
N stage		
N0	51	63.75%
N+	29	36.25%

All the included patients presented with a lateral tongue lesion: 48 (60%) patients had a lesion on the right lateral border whereas the remaining 32 (40%) patients presented with a lesion on the left lateral border of the tongue. Partial glossectomy was done in 58 (72.5%) patients while hemiglossectomy was performed in the remaining 22 (27.5%) patients. Due to severe trismus, mandibulotomy was done in five (6.25%) patients as an approach for surgical resection of tongue lesion; the transoral approach was used in the remaining patients. END dissection was performed in all patients; 38 (47.5%) patients underwent level I to V neck dissection, 24 (30%) patients underwent level I to III neck dissection, and 18 (22.5%) patients underwent level I to IV neck dissection.

After END, pathologically proven occult neck node metastases were seen in 29 out of 80 (36.25%) patients. The mean DOI was 5.1 ±2.2 mm. The DOI was ≤5 mm in 31 (38.75%) patients compared to 49 (61.25%) patients with a DOI >5 mm. A DOI >5 mm was significantly associated with occult neck node metastasis (p=0.04) (Table. 2).

**Table 2:** Correlation of DOI with occult neck node metastases DOI: depth of invasion; CI: confidence interval

DOI	Neck metastasis				Relative risk (95% CI)	P-value
	Yes		No			
	Frequency (n)	Percentage (%)	Frequency (n)	Percentage (%)		
≤5 mm (n=31)	7	22.6	24	77.4	Reference	0.043
>5 mm (n=49)	22	44.9	27	55.1	1.41 (1.03–1.91)	

Patients with a DOI >5 mm had a relative risk of occult neck node metastasis estimated at 1.41. No significant relationship of occult neck node metastasis with age, gender, risk factors, and tumor grade was identified.

## Discussion

The DOI is an important predictor of occult cervical node metastasis, as reported in the literature by multiple authors. The decision to perform END is based on this criterion [6,10-15]. However, the existing data is heterogeneous, and there are huge differences between studies regarding the accuracy of the DOI measurement and its definition. Also, different subsites have been included in these studies, making the results unreliable.

Confusing the DOI with tumor thickness (TT) in the literature has resulted in differing cutoff values of the DOI regarding the decision to perform END [10,11,16]. As the DOI compensates for ulcerative and exophytic tumors, it is considered a better prognostic factor than TT [17].

The eighth edition of the AJCC guidelines defines the DOI as the distance from the normal basement membrane to the deepest point of invasion by the tumor [7]. This outdated many of the studies [10,18,19]. Furthermore, studies conducted after the guidelines were released show discrepancies and do not establish a specific DOI cutoff value. Faisal et al. suggested a 10-mm DOI threshold for consideration for END; Tam et al. suggested a value of 7.25 mm, whereas Kozak et al. did not specify any cutoff value in their study [6,14,20]. In contrast, van Lanschot et al. suggested a 4-mm DOI cutoff value for performing END [13].

Our study showed a significant increase in the risk of occult cervical metastasis in patients with a DOI >5 mm, which is in line with available data. In 2016, Loganathan et al. established a significant association between a DOI >5 mm and the rate of occult metastasis [8]. Similarly, Kumar et al. also showed a significantly increased risk of occult metastasis in patients with a DOI >5 mm and recommended END [9]. On the other hand, a study by Faisal et al. showed 23% occult metastasis in patients with a DOI <5 mm, and they recommend END even in patients with very early tumors [6].

In this study, we focused on a cohort of patients with OTSCC with tumor size measuring ≤4 cm. The risk of bias was managed by using the standard protocol for measuring DOI as defined by the eighth edition of AJCC guidelines [7]. The reporting of all the specimens was done by a specialized head and neck pathologist.

The single-center, retrospective design should be considered as a limitation of this study. A prospective, multi-center study with improved measuring tools and standardized protocols to investigate the correlation of radiological modalities with the histopathological determination of the DOI is the way forward.

## Conclusions

The DOI is an important predictive factor for occult neck metastasis in OTSCC. Our study confirms that the rate of occult neck node metastasis is significantly associated with the DOI. Patients with a DOI >5 mm were 1.41 times more likely to have a neck metastasis than those having a DOI ≤5 mm. Therefore, END should be performed in patients with a DOI >5 mm. Going forward, we recommend a prospective multi-center study investigating the correlation of radiological modalities with the histopathological determination of the DOI.
